# Design of the WHIP-PD study: a phase II, twelve-month, dual-site, randomized controlled trial evaluating the effects of a cognitive-behavioral approach for promoting enhanced walking activity using mobile health technology in people with Parkinson-disease

**DOI:** 10.1186/s12883-020-01718-z

**Published:** 2020-04-20

**Authors:** Kerri S. Rawson, James T. Cavanaugh, Cristina Colon-Semenza, Tami DeAngelis, Ryan P. Duncan, Daniel Fulford, Michael P. LaValley, Pietro Mazzoni, Timothy Nordahl, Lisa M. Quintiliani, Marie Saint-Hilaire, Cathi A. Thomas, Gammon M. Earhart, Terry D. Ellis

**Affiliations:** 1 Program in Physical Therapy, Washington University in St. Louis School of Medicine, St. Louis, MO USA; 2 Department of Physical Therapy, University of New England, Portland, ME USA; 3 Department of Physical Therapy and Athletic Training, Sargent College of Health and Rehabilitation Sciences, Boston University, Boston, MA USA; 4 Department of Neurology, Washington University in St. Louis School of Medicine, St. Louis, MO USA; 5 Department of Occupational Therapy, Sargent College of Health and Rehabilitation Sciences, Boston University, Boston, MA USA; 6 School of Public Health, Boston University, Boston, MA USA; 7 Department of Medicine, Section of General Internal Medicine, Boston University, Boston, MA USA; 8 Department of Neurology, Parkinson’s Disease and Movement Disorders Center, Boston University, Boston, MA USA; 9 Department of Neuroscience, Washington University in St. Louis School of Medicine, St. Louis, MO USA

**Keywords:** Parkinson disease, Exercise, Mobile health, RCT, Walking, Self-efficacy, Cognitive behavioral training

## Abstract

**Background:**

Parkinson disease (PD) is a debilitating and chronic neurodegenerative disease resulting in ambulation difficulties. Natural walking activity often declines early in disease progression despite the relative stability of motor impairments. In this study, we propose a paradigm shift with a “connected behavioral approach” that targets real-world walking using cognitive-behavioral training and mobile health (mHealth) technology.

**Methods/design:**

The Walking and mHealth to Increase Participation in Parkinson Disease (WHIP-PD) study is a twelve-month, dual site, two-arm, randomized controlled trial recruiting 148 participants with early to mid-stage PD. Participants will be randomly assigned to connected behavioral or active control conditions. Both conditions will include a customized program of goal-oriented walking, walking-enhancing strengthening exercises, and eight in-person visits with a physical therapist. Participants in the connected behavioral condition also will (1) receive cognitive-behavioral training to promote self-efficacy for routine walking behavior and (2) use a mHealth software application to manage their program and communicate remotely with their physical therapist. Active control participants will receive no cognitive-behavioral training and manage their program on paper. Evaluations will occur at baseline, three-, six-, and twelve-months and include walking assessments, self-efficacy questionnaires, and seven days of activity monitoring. Primary outcomes will include the change between baseline and twelve months in overall amount of walking activity (mean number of steps per day) and amount of moderate intensity walking activity (mean number of minutes per day in which > 100 steps were accumulated). Secondary outcomes will include change in walking capacity as measured by the six-minute walk test and ten-meter walk test. We also will examine if self-efficacy mediates change in amount of walking activity and if change in amount of walking activity mediates change in walking capacity.

**Discussion:**

We expect this study to show the connected behavioral approach will be more effective than the active control condition in increasing the amount and intensity of real-world walking activity and improving walking capacity. Determining effective physical activity interventions for persons with PD is important for preserving mobility and essential for maintaining quality of life. Clinical trials registration NCT03517371, May 7, 2018.

**Trial registration:**

ClinicalTrials.gov: NCT03517371. **Date of registration:** May 7, 2018. **Protocol version:** Original.

## Background

Parkinson disease (PD) is one of the most disabling chronic health conditions affecting older adults globally [1]. Advances in medical and surgical management of PD have increased lifespans but have not effectively altered the progressive decline in physical function and quality of life accompanying the disease [2, 3]. Given that the prevalence of PD is expected to double to nine million by 2030 [1], identifying effective ways to improve function, slow decline and prevent or reduce disability remains of utmost importance to society [4].

Difficulty with ambulation in PD has been described as a “clinical red flag” signaling emerging disability [3]. A decline in walking function precedes limitations in other gait-dependent activities (e.g., housework, yard work, dressing, traveling) leading to greater disability and reduced quality of life [5, 6]. This phenomenon was captured in a multi-center, natural history, longitudinal study in PD (*n* = 266) which revealed steeper trajectories of decline in gait speed (ten-meter walk test) and gait-related balance over a two-year period compared to other activity level measures [6]. A significant decrease (12%) in number of steps and a 40% reduction in moderate intensity minutes (> 100 steps per minute) over one-year has been described despite the relative stability of motor impairments [7]. A reduction in amount of walking has also been reported early in the diagnosis when motor impairments are mild [8]. Taken together, these results suggested that persons with PD experienced a decline in community walking that was not fully explained by worsening of motor severity.

Interventions targeting walking, the most rapidly deteriorating contributor to disability, may have the greatest impact on slowing the progression of disability in PD [3, 6, 8]. Rehabilitation interventions have traditionally targeted gait-related impairments with the expectation that gains would translate into greater participation in real-world activities. However, the evidence suggests this does not occur [9, 10]. We propose a paradigm shift in which the primary target of intervention is real-world walking behavior, as greater walking activity could preserve walking capacity and slow disability.

Rehabilitation interventions for people with neurological conditions do not typically contain elements that explicitly promote long-term engagement in physical activity such as walking [11]. In our analysis of 266 persons with PD, the primary factors limiting engagement in walking were psychological (e.g., low self-efficacy and poor outcome expectation) rather than physical (e.g., motor impairments) [12, 13]. Without cognitive-behavioral training to develop self-efficacy, gains dissipate and outcomes are compromised [11].

To target self-efficacy we will be employing a “connected behavioral approach” that includes essential elements of cognitive-behavioral training such as identifying unhelpful thoughts, goal setting, action planning, tailored instruction, and feedback reinforcing desired behavior [14]. The connected behavioral approach will be provided by a physical therapist during in-person visits and reinforced via a mobile health software application (mHealth app) that remotely connects participants to therapists following in-person sessions [15]. Our recent PD pilot study revealed that a one-year exercise program rooted in this approach was successful in increasing walking activity and walking capacity among those who were least active [16]. Building on this work, we will conduct a two-arm clinical trial to determine if our connected behavioral approach is more effective than an active control condition in achieving one-year positive outcomes related to walking behavior. To do so, we will recruit less active individuals with early to mid-stage PD and employ a comparator that resembles our intervention but without the cognitive-behavioral and connected mHealth elements [17]. Both conditions contain dynamic walking routines and walking enhancing exercises delivered by physical therapists.

We hypothesize the connected behavioral approach will be more effective than the active control condition in increasing and sustaining real-world walking activity and improving walking capacity among persons with PD over a one-year period. The first study objective is to assess the change in real-world walking activity from baseline to 12 months in the overall amount (number of daily steps) and the amount of walking activity that meets or exceeds an established threshold for moderate intensity (number of minutes per day with greater than 100 steps) recorded over a seven-day period [18, 19]. We hypothesize participants in both conditions will improve; however, those in the connected behavioral condition will have greater improvement in daily steps and moderate intensity minutes compared to the active control condition. The second objective is to assess change in walking capacity over 12 months using the six-minute walk test (6MWT) and ten-meter walk test (10MWT). We hypothesize participants in the connected behavioral condition will have greater improvement in walking capacity compared to the active control condition. The final objective is to identify the potential mechanisms that account for observed improvements in the connected behavioral condition. We hypothesize change in self-efficacy will mediate change in walking activity and that change in walking activity will mediate change in walking capacity over one year.

## Methods/design

### Ethical approval and trial registration

The study is approved by the Boston University Institutional Review Board (IRB), the reviewing IRB and single IRB of record. Modifications to the protocol will be sent to study team members and the Data Safety and Monitoring Board (DSMB) as needed for comments, then submitted to the IRB for approval. The study team will be notified of new procedures after IRB approval. The WHIP-PD trial was registered with ClinicalTrials.gov on May 7, 2018 (NCT03517371).

### Study design and settings

This is a twelve-month, two-arm, single-blinded, randomized controlled trial occurring at two academic sites. Participants will attend evaluation sessions and physical therapy visits at either the Sargent College of Health & Rehabilitation Sciences or Charles River Campus (and satellite locations) at Boston University in Boston, Massachusetts, USA or the Program in Physical Therapy, Washington University (WU) School of Medicine in St. Louis, Missouri, USA. We have adhered to the SPIRIT guidelines/methodology for this manuscript.

### Study population

Based on our experience conducting exercise trials in PD, we established inclusion and exclusion criteria to ensure participants could safely engage in a home/community walking and exercise program (Table 1). The criteria reflect our interest in delivering the intervention to relatively less active, community-dwelling, medically stable individuals with early- to mid-stage PD.

**Table 1:** Inclusion and exclusion criteria

Inclusion criteria	Exclusion criteria
▪ Diagnosis of idiopathic, typical PD according to the United Kingdom Brain Bank Criteria [20] ▪ Modified Hoehn & Yahr stages 1–3 (mild to moderate disease severity) [21] ▪ Live in the community ▪ Able to walk 10 continuous minutes without help from another person ▪ Stable on all PD medications for at least two weeks prior to study entry	▪ Moderately or significantly disturbing freezing episodes during daily walking (score of ≥ 2 on item 7 of the New Freezing of Gait Questionnaire (nFOGq)) [22] ▪ Significant cognitive impairment (i.e., Mini-Mental State Examination (MMSE) score of < 24) [23] ▪ Unstable medical or concomitant illnesses or psychiatric conditions, which in the opinion of the investigators would preclude successful participation ▪ Cardiac problems that interfere with ability to safely exercise (i.e., uncontrolled congestive heart failure, complex cardiac arrhythmias, chest pain or pressure, resting tachycardia (> 120 beats/min), uncontrolled BP (resting systolic BP > 180 mmHg or diastolic BP > 100 mmHg)) ▪ Orthopedic problems in the lower extremities or spine that may limit walking distance (i.e., severe arthritis, spinal stenosis or pain) ▪ Engaged in a walking program for greater than 90 min per week for the past month ▪ Engaged in an exercise regime of moderate intensity for greater than 90 min per week for the past month

### Recruitment

At BU, recruitment will occur primarily through the PD and Movement Disorders Center at the School of Medicine/Boston Medical Center and The Center for Neurorehabilitation (CNR). At WU, recruitment will occur through the Movement Disorders Center at WU School of Medicine. Methods of recruitment include research coordinators contacting potential participants from patient registries at each institution; neurologists or physical therapists identifying potential participants during regular clinical visits; distributing approved flyers to PD support groups, physicians, and physical therapists in the community; approved advertisements in the American Parkinson Disease Association (APDA) newsletter in Massachusetts and Missouri; and posting study information online at ClinicalTrials.gov, Fox Trial Finder, CNR website, and on the APDA’s and Program in Physical Therapy at WU Facebook page.

Research coordinators will conduct a telephone screening with individuals interested in participating to review the inclusion and exclusion criteria listed in Table 1. Individuals who pass the telephone screening will be invited to the local study site to undergo an in-person screening session. Participants will be scheduled for the screening session when they have had no change in their PD medications for at least two weeks. Trained study staff will begin the in-person session by explaining the study in detail and reviewing the informed consent document. The potential participant will be informed that participation in the study is entirely voluntary and will have no effect on any present or future medical or rehabilitation care. The potential participant will be encouraged to ask questions about the study to ensure complete understanding of all study elements and be provided with as much time as they request to review the informed consent and to ask questions. Only when the potential participant has provided full written informed consent will trained study personnel proceed to the in-person screening and subsequent study procedures. Consented participants who pass the in-person screening, and are therefore eligible for the full study, will continue with the baseline evaluation on the same day.

### Randomization and blinding

Block randomization of participants to the two treatment conditions will be completed using the Research Electronic Data Capture (REDCap) Randomization Model [24–26]. A randomized allocation table will be created in Microsoft Excel stratifying by gender (male and female), disease severity as determined using the Modified Hoehn & Yahr scoring (<= 2 and > =2.5) [21], and site (BU and WU). Entries in the table will be sorted using the Excel RAND function (=RAND) to assign a number between zero and one. The table will be uploaded subsequently into REDCap. After confirming eligibility and obtaining consent, participants will be randomized to Treatment Y or Treatment Z by clicking the randomize button in REDCap.

Raters that are blinded to treatment condition will conduct in-person evaluations and administer standardized outcome measures at each assessment. Only unblinded team members will be aware of what Treatment Y and Treatment Z represent. Unblinding of raters will not be permitted in any circumstance unless mandated by DSMB due to safety concerns about the trial.

### Participant timeline

**Fig. 1:**
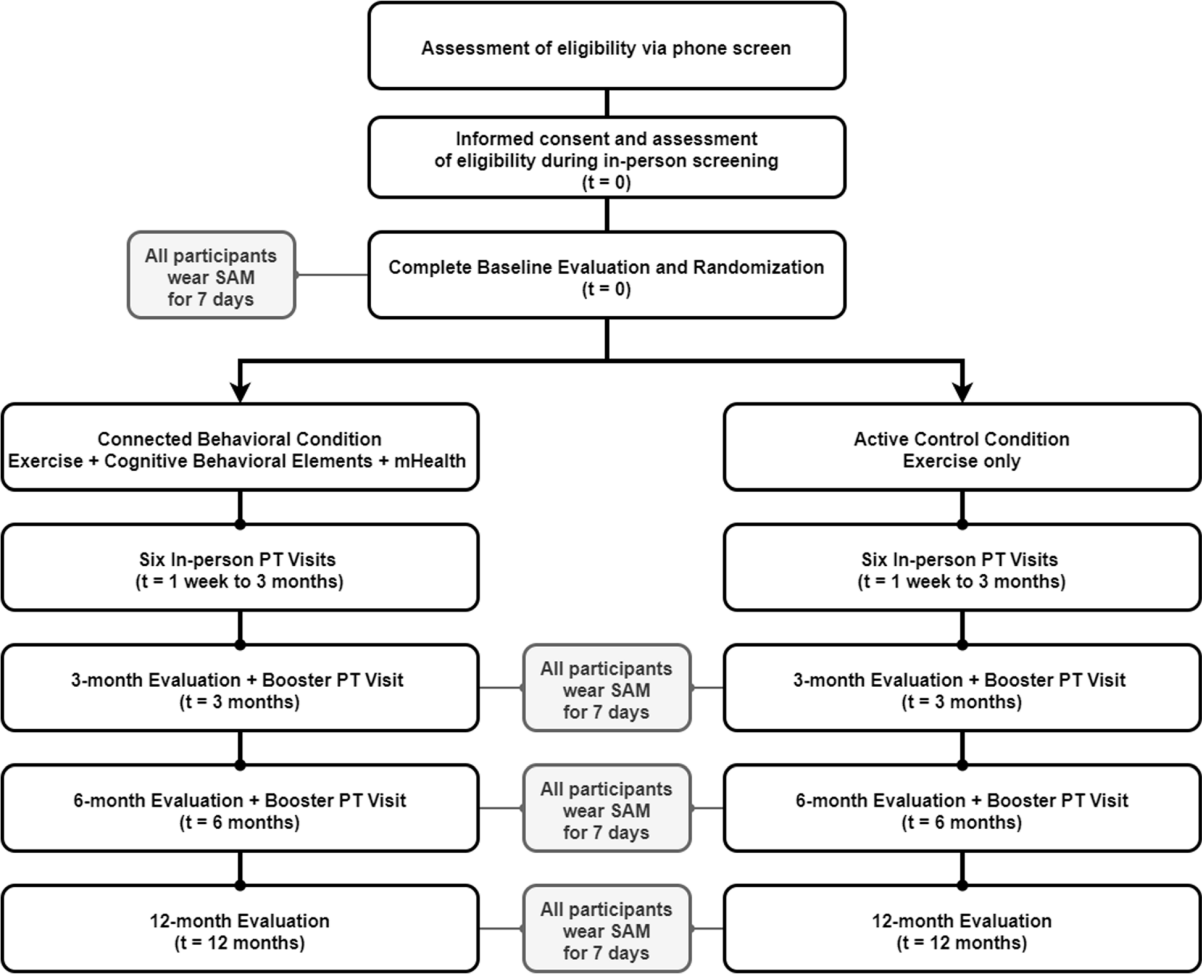
Participant progression through the twelve-month randomized controlled trial. Participants will be randomly assigned to the Connected Behavioral Condition that includes exercise, cognitive behavioral elements, and mobile health (mHealth) technology or the Active Control Condition that includes exercise only

Eligible participants will participate in a baseline evaluation session (approximately 3 h in duration) that will occur on the same day as their successful screening (Fig. 1). Both exercise conditions include eight in-person physical therapy intervention sessions (approximately 30 to 60 min in duration), evaluations at three and six months (approximately 2.5 h in duration), and a final evaluation session at twelve months (approximately 2.5 h in duration). Ideally, evaluations will be scheduled within one to two weeks of the target date for the three-, six-, and twelve-month evaluations. Participants will be tested in their self-reported *on* state during the study evaluations and maintain their medication regimen as directed by their treating neurologists.

### General variables

During the baseline evaluation session, we will collect participant characteristics (i.e., age, gender, race, occupation, education level, living situation, disease specific information including year of PD diagnosis), fall history, medical history, and medications including determining levodopa-equivalent dose (LEDD). The New Freezing of Gait Questionnaire (nFOGq) [22] will be used to verify that freezing episodes, if applicable, do not moderately or significantly interfere with daily walking, and the Mini-Mental State Examination (MMSE) [23] used to ensure absence of significant cognitive impairment. Falls, in which a person comes to rest on the ground, will be tracked during the study and changes in medical history and medications will be recorded at each subsequent evaluation session. Table 2 provides a list of these variables and the primary and secondary outcome variables.

**Table 2:** Data collection schedule

	Baseline	3 Month	6 Month	12 Month
**Patient characteristics**				
Age, Gender	X			
Education level	X			
Living situation	X			
Occupation	X			
Race/ethnicity	X			
**Medical history**				
Comorbidities	X	X	X	X
Falls	X	X	X	X
Medications	X	X	X	X
Parkinson symptom duration	X			
**Primary outcomes**				
Number of steps per day	X	X	X	X
Number of moderate intensity minutes per day	X	X	X	X
**Secondary outcomes**				
Six-minute walk test	X	X	X	X
Ten-meter walk test	X	X	X	X
Self-Efficacy of Walking - Duration	X	X	X	X
Barriers Self-Efficacy Scale	X	X	X	X

### Primary outcome measures

Change in the amount and intensity of daily real-world walking activity between the baseline and twelve-month evaluations are the primary study outcomes. Using the StepWatch™ 4 Activity Monitor (SAM; Modus Health LLC, Edmonds, WA), we will examine daily step counts and moderate intensity minutes (i.e., the number of minutes in which more than 100 steps were taken) recorded over seven consecutive days during the week following each in-person evaluation session. Participants will wear the SAM 24 h per day (except when bathing, showering, swimming). Mean daily steps and moderate intensity minutes will be calculated at each assessment point. The SAM is a small, waterproof, highly durable, self-contained device that is approximately the size of a pager, weighs 38 g, and will be attached using Velcro closures immediately proximal to the lateral malleolus of the leg that is less affected by PD. The SAM records the number of strides taken every minute with the leg of attachment using a combination of acceleration, position, and timing. It is designed for long-term use during daily activities performed in an individual’s customary environment over hours or days without maintenance by the user.

The SAM does not provide feedback to users regarding number of steps taken, thereby reducing its influence on behavior. The SAM has good validity and reliability in older adults and people with PD [27]. Step detection accuracy exceeds 98% both for unimpaired gait and for movement styles that have traditionally been difficult to monitor accurately, such as Parkinsonian shuffling, hemiparetic gait, and dyskinetic gait [28, 29]. The SAM is a more accurate method of assessing adherence to a walking program than self-report measures [30].

### Secondary outcome measures

To examine change in walking capacity over the one-year period, we will use data from the six-minute walk test (6MWT) and ten-meter walk test (10MWT). The 6MWT is a safe, valid, reliable, and responsive measure of the distance, in meters, that a participant walks in a six-minute period [31, 32]. The participant will be instructed to cover as much ground as possible while walking back and forth around two cones placed 30 m apart. Greater distances indicate increased ability for community ambulation. The 10MWT measures gait speed and is a reliable and valid measure for people with PD [33]. The test includes an initial two-meter acceleration phase, followed by six meters of ambulation, and finishes with a two-meter deceleration phase. Only the middle six meters will be timed and the average speed of two trials at a comfortable pace will be computed for analyses.

We will use two measures of self-efficacy. The Barriers Self-Efficacy Scale (BARSE) is a self-administered questionnaire in which participants rate their confidence in exercising ‘in the event that any of the following circumstances were to occur (e.g., weather was very bad, on vacation)’ [34]. Confidence is rated on a 100-point percentage scale comprised of 10-point increments, ranging from 0 to 100% for each of the thirteen items, where 0 equals ‘not confident at all’ and 100 equals ‘highly confident’. Total score is calculated by summing the scores and dividing by 13 (0–100%). Higher scores indicate greater confidence in the ability to exercise. The Self-Efficacy of Walking - Duration (SEW-D) scale is a self-administered questionnaire to measure participants’ beliefs in their ability to successfully walk at a moderately fast pace without stopping for different durations of time ranging from 5 to 50 min [35]. For each of the ten items, participants will indicate their confidence in executing the behavior on a 100-point percentage scale comprised of 10-point increments, ranging from 0% (not at all confident) to 100% (highly confident). The total score will be calculated by summing the score of each item and dividing by 10 (0–100%). Higher scores indicate greater confidence in walking ability.

### Interventions

A physical therapist at each site will be assigned to conduct in-person intervention sessions. The physical therapists have extensive clinical experience in the assessment and rehabilitation of persons with PD. A clinical psychologist (DF) will provide both therapists with additional training in the administration of the cognitive-behavioral approach.

All participants will complete a baseline evaluation session followed by six, 30–60 min intervention visits with the physical therapist. The six intervention visits will be scheduled between the baseline and three-month evaluation sessions. Two booster intervention visits will occur following the three- and six-month evaluation sessions. The final evaluation session will occur twelve-months after the baseline evaluation. While there are no planned interactions among the participants and physical therapists between the six-month booster session and the final twelve-month evaluation, participants may initiate contact with the physical therapist if questions or problems arise.

Participants in both the connected behavioral and active control conditions will receive a customized intervention consisting of a walking program and strengthening exercises designed to enhance walking ability. The walking program consists of two parts: 1) dedicated bouts of walking; and 2) gradually increasing amount (number of minutes) of walking and frequency (days per week) of walking. The goal for the dedicated walking bouts consists of continuous walking in the community or on a treadmill for 30 min, five times per week. If necessary, participants will start with a minimum of 10 min and gradually increase to 30 consecutive minutes of walking during the first three months of the intervention. Exercises designed to enhance walking ability will be chosen for each participant from a list of exercises modified from previous work [16] (Table 3). The type and intensity of prescribed exercises will be customized for each participant based on the results of their baseline evaluation. To reduce barriers to implementing the program at home, only exercises that do not require specialized equipment will be utilized. Participants will work toward the goal of performing at least five exercises on five days per week by the time they reach the three-month evaluation session. During the first three months, exercises will be progressed by the physical therapist during in-person visits to maintain an appropriate level of challenge for each individual.

**Table 3:** Exercise program for participants in connected behavioral and active control conditions

Level 0	Level 1	Level 2	Level 3	Level 4
**Stretch:** Hamstring	**Squat:** Sit to Stand	**Squat:** Partial Squat	**Squat:** Full Squat	**Squat:** Single Leg Squat
**Stretch:** Hip Flexor	**Lunge:** Reverse Lunge (with support)	**Lunge:** Reverse Lunge (no support)	**Lunge:** Walking Lunges	**Lunge:** Walking Lunges (with arms)
**Stretch:** Supine Trunk Rotation	**Lateral Hip Strength:** Side Leg Lifts (with support)	**Lateral Hip Strength:** Side Leg Lifts (no support)	**Lateral Hip Strength:** Lateral Lunge	**Lateral Hip Strength:** Step Back Cross Lunge
**Stretch:** Calf	**Heel Raise:** Both Legs (with support)	**Heel Raise:** One Leg (with support)	**Heel Raise:** Both Legs (no support)	**Heel Raise:** One Leg (no support)
	**Step-Ups:** Alternating Foot Taps	**Step-Ups:** One Foot Step-Up	**Step-Ups:** Both Feet Step-Up	**Step-Ups:** Step-Up with Knee Lift
	**Bridge:** Bridge (hands on floor)	**Bridge:** Bridge (arms elevated)	**Bridge:** Bridge with Single Leg Extension	**Bridge:** Single Leg Bridge
	**Push-Up:** Wall Push-Up	**Push-Up:** Counter Push-Up	**Push-Up:** Modified Push-Up	**Push-Up:** Standard Push-Up
	**Multidirectional Stepping:** Front/Side Steps	**Multidirectional Stepping:** Front/Side Floor Taps	**Multidirectional Stepping:** Front/Side/Cross Cup Taps	**Multidirectional Stepping:** Full Circle Cup Taps

Modifications to the exercise program for a given participant will be permitted in the event of a new medical condition or change in health status. Modifications may include temporarily suspending all or part of the exercise and walking program; prescribing alternative, less challenging exercises; reducing the number of sets or reps of already prescribed exercises; or decreasing the intensity of the walking program. Participants may withdraw voluntarily at any time, and principal investigators can withdraw any participant from the study if a change in medical status compromises safe participation.

Participants in both conditions will maintain their standard of care with their medical team during the trial. Changes in medications will be permitted while participants are enrolled in the study and recorded at evaluation sessions. Participants will be instructed to indicate their participation in any additional exercise or recreational programs outside those prescribed in the intervention.

### Connected behavioral condition

The connected behavioral condition includes elements of cognitive-behavioral training (CBT) that will be delivered in-person and reinforced via the mHealth app. CBT emphasizes participant engagement in managing a person’s health condition through increasing self-efficacy. During the eight physical therapy visits, the physical therapist will deliver cognitive-behavioral content that includes reinforcing the benefits of exercise; establishing value-based goals; introducing connection between thoughts, mood, and exercise behavior (e.g., adherence); identifying unhelpful thoughts that serve as barriers to physical activity; introducing thought challenging (e.g., weighing evidence for and against thoughts, constructing new, balanced thoughts); and overcoming barriers to physical activity and strategies to prevent relapse (i.e., inactivity) (Table 4). The physical therapist will work collaboratively with each participant using CBT principles to set specific, incremental, and attainable walking and exercise goals. Each participant will have a detailed action plan that includes what (which exercises, duration of walking), how (appropriate technique), when (time of day, days per week), and where (community, mall) they will engage in their walking and exercise program.

**Table 4:** Connected behavioral condition: Cognitive-behavioral training (CBT) and mHealth app integration

**Major Content and Activities**		
Each session builds upon the content of the previous session. Session format includes: (1) review of previous session and collaborative agenda setting; (2) discussion of success and challenges with program; (3) revisiting value-based, personal goals; (4) introduction of new cognitive-behavioral training (CBT) content through discussion; and (5) building exercise program in Wellpepper mHealth app and establishing new goals.		
	**Cognitive-Behavioral Features**	**Connected Health Features in App**
Sessions 1 & 2 Introduction to CBT; Initiate exercise program in app and establish value-based goals	▪ Discuss personal, value-based goals and add to Wellpepper platform ▪ Identify the pros and cons of exercise & multi-level factors that affect physical activity ▪ Discuss facilitators and barriers to exercise and set an action plan ▪ Introduce relationship between situations, thoughts, and behavior	▪ Instruct in use of tablet & mHealth app ▪ Develop walking program and video exercise catalog in app ▪ Discuss rating challenge in app and how to communicate with the physical therapist ▪ Introduce self-monitoring in app through review of calendars and graphs
Sessions 3 & 4 Challenge your thoughts, Balance your thinking and progressing the program	▪ Examine thoughts around exercise and how they are linked to situations/behaviors ▪ Introduce thinking traps and discuss potential impact on exercise behaviors ▪ Introduce thought challenging ▪ Incorporate thought challenging into self-monitoring	▪ Participant encouraged to demonstrate self-monitoring, self-assessment through adherence reports in app ▪ Additional video exercises recorded as program progressed to maintain challenge ▪ Review walking and exercise goals; revise goals as needed
Sessions 5 & 6 Identifying high risk situations and developing strategies to cope; Staying healthy	▪ Consider high risk situations when it will be difficult to stick with exercise program ▪ Develop both preventative and coping strategies to manage ▪ Devise plan for staying healthy	▪ Re-assess appropriate challenge level of program ▪ Review goals and discuss readiness for gradual increase in amount of walking and dose of exercise ▪ Discuss communication plan in app through messaging
Sessions 7 & 8 (Boosters)	▪ Reinforce benefits of program ▪ Address additional cognitive barriers ▪ Discuss/review relapse cycle and relapse prevention strategies ▪ Revisit unhelpful thoughts, cognitive restructuring, self-monitoring	▪ Discuss how to safely re-engage in the exercise program if set-backs occur ▪ Discuss adaptations to the program if set-backs occur ▪ Reinforce self-monitoring of progress ▪ Discuss how to self-monitor, determine how/when to adjust exercise level and dose over time

The mHealth app links participants to a proprietary, web-based “patient engagement platform” (Wellpepper, Inc., Seattle, WA) designed to promote patient self-management and remote connection to personal healthcare providers. The Wellpepper mHealth app is available through the app store on both Android and Apple devices. At the first physical therapy session, the physical therapist will set up the mHealth app on the participant’s personal device (phone or tablet) and provide the necessary instruction in its use. If a participant does not have an appropriate device or Wi-Fi in their home, a tablet with cellular service and the mHealth app already installed will be provided. The physical therapist will take video recordings of the participant performing their initially prescribed strengthening exercises while being instructed in proper technique. The videos will be uploaded to the mHealth app for the participant to access at home. Video recordings of new exercises will be added during subsequent intervention sessions. Participants will be reminded to complete their exercises through automated notifications on their device. To encourage self-monitoring and feelings of mastery, participants will receive visual feedback/rewards (i.e., fireworks image) when intervention goals are attained.

The mHealth app also provides a secure, HIPAA compliant means for participants to connect to the physical therapist following in-person sessions. The increased connectivity will provide participants with additional support for effectively interpreting their physiologic and affective responses to their walking and exercise program and for developing greater self-efficacy. Participants can use the messaging feature of the mHealth app to ask clarifying questions about the exercise program, to report any barriers or problems that may have arisen, seek advice, or receive confirmation of success.

The Wellpepper software platform also serves as a web-based participant management site for the physical therapist. This site permits the physical therapist to monitor adherence, exercise difficulty ratings, survey responses, and adapt the walking and exercise program remotely at any time. If the participant has not logged into the Wellpepper platform for seven consecutive days, the physical therapist will be automatically notified. The physical therapist will reach out to the participant and adjust the program if needed or address any barriers to facilitate re-engagement.

### Active control condition

Neither cognitive-behavioral training nor the mHealth app will be provided to participants in the active control condition. Participants instead will receive their individualized walking and exercise program with instructions and pictures printed on paper and provided in a binder. Instructions on progressing the exercises to maintain an appropriate level of challenge will be included. Participants will receive a phone number to reach the physical therapist should they have any further questions or concerns about their exercise program. To monitor exercise adherence, participants in the active control condition will have a journal to indicate which days they completed the exercises. Participants will bring the completed exercise journals with them to their in-person intervention visits.

### Power and sample size estimate

In our pilot study, low activity participants in the connected behavioral condition had 6028 (SD 1046) steps per day at baseline and 6918 (1900) at 12 months. Participants in the active control condition had 6330 (SD 560) steps per day at baseline and 6788 (SD 1636) at 12 months [16]. Using the Two-Sample T-Test Allowing Unequal Variance Procedure in PASS Power Analysis and Sample Size software [36], group sample sizes of 61 participants per group will be needed to achieve 80% power, ɑ = .05. To account for 20% drop out rate, 74 participants will need to be recruited per group for a total of 148 participants. This sample size will provide sufficient power to also detect differences in number of minutes of moderate intensity steps collected over seven days via the SAM. With 74 participants per group we will have 86% power to detect an effect size of 0.5 in number of minutes of moderate intensity steps.

### Data collection and management

Prior to enrollment, research team members participating in data collection will meet in-person to ensure rater reliability and consistency of assessment administration between sites. Participant retention methods will include making phone calls to remind participants of their upcoming visits and modifying the exercise program if a participant experiences a change in physical health.

To promote data quality, we will be using a REDCap database hosted at Boston University. REDCap is a secure, web-based software platform designed to support data capture for research studies [24–26]. A data entry assistant initially will enter data collected on paper into REDCap and each questionnaire will be marked as “unverified”. A second data entry assistant will then check that the data were entered correctly and mark each questionnaire as “complete”. Each data entry assistant will receive REDCap training and watch instructional videos housed on the REDCap website. The raters administering the evaluation questionnaires will check for data completion during the evaluation sessions.

Only de-identified data will be entered in the REDCap database and de-identified source documents will be housed at BU and WU in locked filed cabinets in locked offices. All data and data monitoring will be kept strictly confidential according to HIPAA regulations. The master code linking study IDs to identifiable information will be housed in a password-protected file with access restricted to essential study staff who will log in using a two-factor authentication process.

Following each seven-day recording period, a participant’s SAM data (identified only by a study identification number) will be downloaded from the monitor to a personal password protected iPAD using the manufacturer’s software. Raw SAM data files from each day of recording subsequently will be uploaded to a password protected iCloud site for visual inspection by a blinded research team member to ensure their integrity. Primary outcome variables then will be calculated from valid recording days and uploaded to the REDCap database. Data collected through the Wellpepper platform is encrypted in transit and at rest per HIPAA standards. Wellpepper runs in a virtual private cloud using MySQL and MongoDB databases. For study participants using the mHealth app, Wellpepper requires strong passwords, that users change passwords every 90 days, and that passwords are not reused. After the study, data stored by Wellpepper will be retained by the study principal investigators and not by Wellpepper. Exercise videos, housed on Wellpepper’s private cloud, will be destroyed at the end of the study.

### Statistical analysis plan

Our primary aim is to determine effectiveness of the connected behavioral condition for improving walking behavior in comparison to an active control condition. We will conduct the main analyses on the full sample, comparing the change from baseline to one year in mean number of steps per day and number of minutes per day in which participants walk greater than 100 steps per minute (i.e., moderate intensity minutes). The unadjusted treatment comparison will be determined using a Satterthwaite unpooled t-test to allow for unequal variance in treatment groups. Study site and any baseline variables that differ between conditions will be controlled for as covariates in additional multiple linear regression analyses (equivalent to the analysis of covariance). The two-sided .05 level will be used for significance in all analyses. Strength of effect size estimators (percent change and Cohen’s d effect sizes) will be calculated for all measures.

As a second approach for examining participation outcomes, we will use hierarchical mixed effects regression models for repeated-measures (longitudinal data) analysis of the SAM data at baseline, three-, six-, and twelve-months. These analyses model the correlation between repeated measures from the same participant by incorporating subject as a random effect in the model. The hierarchical mixed effects regression approach has several advantages over traditional repeated measures analysis of variance, including ability to incorporate data from participants with incomplete follow-up in the analysis without imputation (e.g., allowing data from participants with twelve-month but not six-month data to be included) and more flexibility in modeling the correlation between repeated observations. The models will include main effect terms for intervention group and time and the interaction between group and time. The interaction will be used to test for the intervention effect. This approach controls for potential confounding variables, or other variables strongly associated with outcomes, as covariates.

We will use a similar approach to the primary analysis described above to analyze the effect of the intervention on secondary outcomes of walking capacity (6MWT and 10MWT). We will again examine change between baseline and one-year and use hierarchical mixed regression models to analyze changes over time between the two conditions. Each hypothesis will be tested two-sided (level of significance of .05). All statistically significant differences between groups at baseline will be included as covariates.

For our last objective, we will examine mediating variables from the evaluation before the outcome assessment. That is, SAM data at six-months will be adjusted for three-month self-efficacy score, and the twelve-month SAM data adjusted for the six-month self-efficacy score. A separate analysis will examine walking capacity data adjusted for SAM data (amount of walking) using the same approach. Both regression models will allow repeated outcome assessments and evaluation of the role of these potential mediating variables. Likelihood ratio tests will be used to compare the model including the lagged mediator as a predictor with the model not including the lagged mediator. Mediation analyses will follow procedures as described in Valerie and VanderWeele [37].

Distributions will be examined to determine the need for data transformation, winsorizing, or nonparametric analyses. Multiple imputation will be used to address any missing values due to participant withdrawals or incomplete assessments. Multiple imputation will be used to create complete datasets for use in all analyses other than mixed models, where the restricted maximum likelihood procedure produces approximately unbiased estimates so long as data are missing at random [38] and is less sensitive to missing data than analyses restricting to complete data [39].

### Monitoring

The study team will conduct a quarterly review of recruitment, enrollment, and data management to ensure the study is progressing in a timely manner. The Data Safety and Monitoring Board (DSMB) will meet twice annually by teleconference call to review study progress, data quality, and participant’s safety and overall risk to benefit ratio. Safety reports will be sent to the safety officer (SO) twice a year and will include a detailed analysis of study progress, data, and safety issues. The DSMB Charter provides a detailed list of the DSMB/SO responsibilities, which include reviewing the research protocol, informed consent documents, and plans for data safety and monitoring; advising on the readiness of the study staff to initiate recruitment; evaluating the progress of the trial, including periodic assessments of data quality and timeliness, recruitment, accrual and retention, participant risk versus benefit, performance of the trial sites, and other factors that can affect study outcome; consideration of factors external to the study when relevant information becomes available, such as scientific or therapeutic developments that may have an impact on the safety of the participants or the ethics of the trial; reviewing study performance, making recommendations and assisting in the resolution of problems reported by the principal investigators; protecting the safety of the study participants; reporting on the safety and progress of the trial; making recommendations concerning continuation, termination or other modifications of the trial based on the observed beneficial or adverse effects of the treatment under study; ensuring the confidentiality of the study data and the results of monitoring; and commenting on any problems with study conduct, enrollment, sample size, and/or data collection.

Bi-annual reports submitted to the DSMB two weeks prior to the twice-yearly teleconference calls will include information regarding recruitment, enrollment and flow of participants through the study, missing data and any adverse events, participant concerns and any unexpected problems. The DSMB will provide a written summary of record for each of their meetings and will communicate any areas of concern to the principal investigators, the IRB, and the National Institutes of Health as appropriate.

Any serious adverse events will be reported to the principal investigators within 48 h of discovery. Any serious adverse events possibly or definitely related to the intervention will be reported to the IRB of record and the DSMB within five days of discovery and will trigger an immediate review to determine what changes need to be made and whether the study should continue or conclude.

### Harms

The risks of participating in this trial will be minimal, given that the connected behavioral and active control conditions feature a moderate intensity walking and exercise program which is customized based on the exercise tolerance of each participant. The program was found to be safe in our pilot study as well as in other studies of people with PD [16]. In addition, a physical therapist will prescribe and instruct the elements of the program to the participants (e.g., how and when to progress amounts of walking and exercises) and will adapt the program to meet their specific needs.

Participant safety will be monitored throughout the study. If participants have an injury of any kind, experience a change in health condition, need medical attention, are hospitalized, or experience a fall, they will be asked to contact the research team within 48 h using a designated phone number provided at each site. Participants will be instructed by study staff about when to report changes in their health and how falls are defined during their baseline evaluation. All participants will be provided with a printed calendar to indicate the date on which a fall or health event occurs. Participants who experience a fall in which they come to rest on the ground will be asked to call the designated phone number and the study team will conduct a fall phone interview about the nature of the fall. Participants will turn in their falls calendars when they attend their evaluation sessions.

When an adverse event is reported, staff will complete an adverse event form and inform other study staff about non-serious events on monthly conference calls. The adverse events will be classified using following terms: expected or unexpected (e.g., falls and musculoskeletal injuries are expected in this population); definitely related, possibly related or unrelated; mild, moderate, or severe; resolved or ongoing. Research coordinators will follow up and track adverse events while the participant is enrolled until the event status is resolved.

## Discussion

People living with PD typically experience a decline in their ambulation or walking abilities. Interventions that target walking behaviors exclusively in this population are lacking and almost never include training to promote long-term self-management of physical activity and exercise [40]. Our previous analysis of people with PD revealed that physical activity was limited by low self-efficacy for engagement in a walking-oriented intervention program and low expectations for a successful outcome [16]. Without cognitive-behavioral training to develop self-efficacy, gains dissipate and outcomes are compromised [11]. Given the importance of physical activity in reducing disability and mitigating PD progression, we must integrate effective approaches to sustain activity over the long-term.

Our objectives here include determining if a connected behavioral approach will be more effective than an active control condition in increasing real-world walking activity. Participants in both conditions will undergo four evaluations at baseline, three-, six- and twelve-months, each lasting two-three hours in length. Participants will receive a tailored exercise program that consists of walking and strengthening exercises and eight in-person visits with a trained physical therapist. The connected behavioral approach includes cognitive-behavioral training to increase self-efficacy combined with management of the walking and exercise program via a mHealth app that encourages self-monitoring and provides a remote connection to the physical therapist. The exercise program for the active control condition is delivered in paper form and participants record their exercises on paper.

The primary and secondary objectives for the WHIP-PD study include comparing differences in walking activity and walking capacity between groups over a one-year period. Parameters for walking activity will be derived from a SAM worn over a seven-day period following each evaluation session and include number of steps per day and number of moderate intensity minutes. Parameters for walking capacity will be derived from the 6MWT and 10MWT. We will also determine if change in self-efficacy mediates change in walking activity and if change in walking activity mediates change in walking capacity for the connected behavior condition. We will also track falls and injuries during the study period to monitor the safety of the intervention.

The data collected from this study will increase our understanding of the intervention components necessary to increase walking activity among people living with PD. While previous studies have indicated that exercise is favorable in reducing motor impairments in PD, we anticipate that this novel, connected behavioral approach for targeting real-world walking will provide greater benefits to participants and inform future interventions to encourage walking.

## Data Availability

The final dataset will be readily accessible only to study team members. Requests from investigators outside of the study team will be handled on a case by case basis and granted as permitted under our IRB approval conditions. Study results will be shared through peer-review manuscripts and conference presentations. Authorship will include those who make substantial contributions to the conception, design, acquisition, analysis, and/or interpretation of the work. Authors will also be involved in drafting and revising the manuscripts, as well as final approval of the manuscripts.

## Abbreviations

6MWT: Six-minute walk test
10MWT: Ten-meter walk test
APDA: American Parkinson Disease Association
BARSE: Barriers Self-Efficacy Scale
BP: Blood pressure
BU: Boston University
CBT: cognitive-behavioral training
CNR: The Center for Neurorehabilitation
DF: Daniel Fulford, clinical psychologist
DSMB: Data Safety and Monitoring Board
HIPAA: Health Insurance Portability and Accountability Act
IRB: Institutional Review Board
LEDD: Levodopa-equivalent dose
mHealth app: Mobile health software application
MMSE: Mini-mental state examination
nFOGq: New Freezing of Gait Questionnaire
PASS: Power Analysis and Sample Size
PD: Parkinson disease
RAND: Random
RCT: Randomized controlled trial
REDCap: Research Electronic Data Capture
SAM: StepWatch™ 4 Activity Monitor
SD: Standard deviation
SEW-D: Self-Efficacy of Walking – Duration
SO: Safety officer
Wellpepper: Web-based “patient engagement platform”
WHIP-PD: Walking and mHealth to Increase Participation in Parkinson Disease
Wi-Fi: Wireless fidelity
WU: Washington University School of Medicine in St. Louis
